# Supplementary figures and images for: Health determinants among refugees in Austria and Germany: A propensity-matched comparative study for Syrian, Afghan, and Iraqi refugees
Source: PLoS One. 2021 Apr 28;16(4):e0250821. doi: 10.1371/journal.pone.0250821 (PMC8081210; doi:10.1371/journal.pone.0250821)

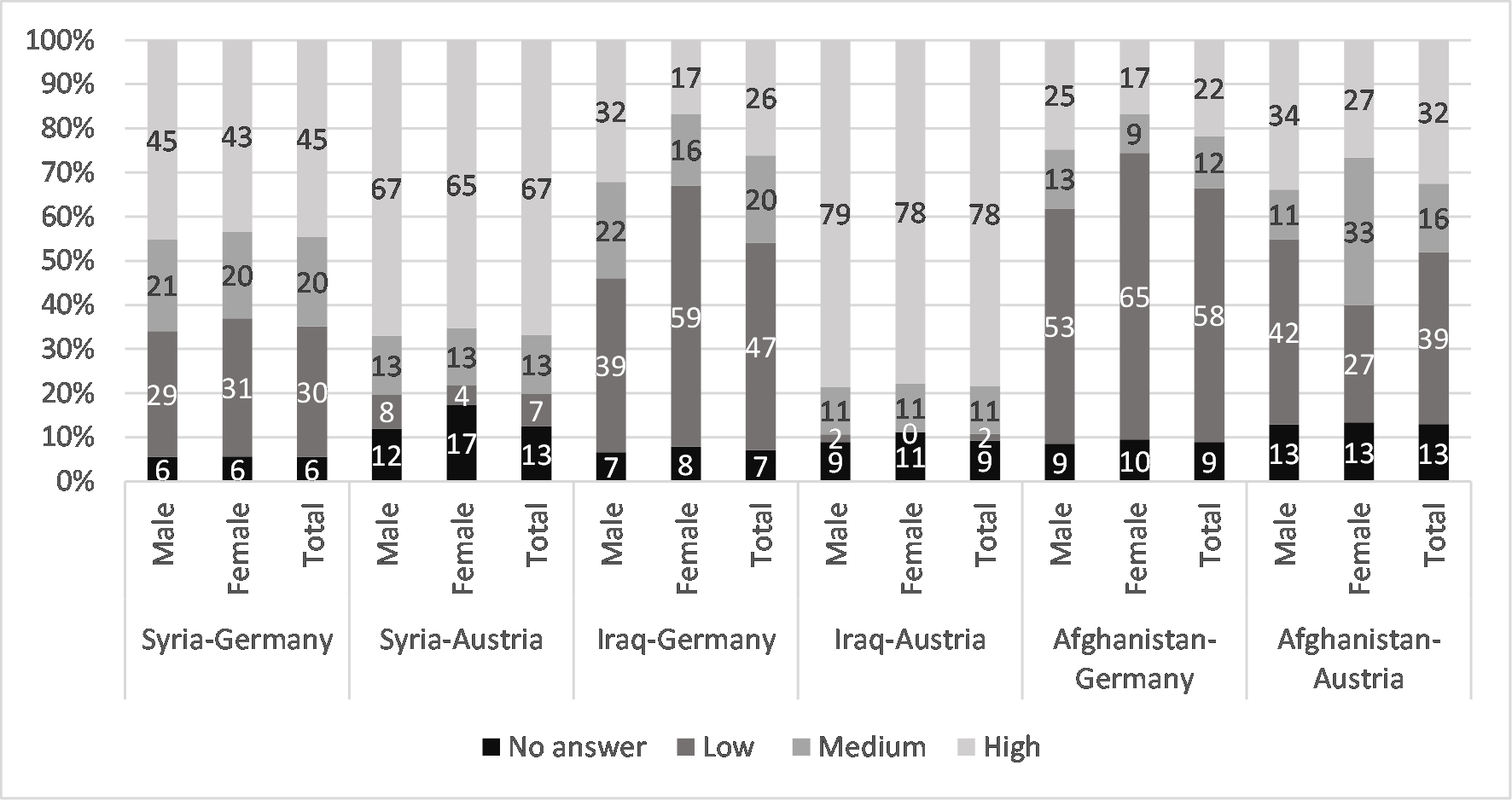

Supplement: S1 Fig — Sources: IAB-BAMF-SOEP 2016, ReHIS. (TIF) [file pone.0250821.s001.tif]
