# Supplementary material for: Health determinants among refugees in Austria and Germany: A propensity-matched comparative study for Syrian, Afghan, and Iraqi refugees
Source: PLoS One. 2021 Apr 28;16(4):e0250821. doi: 10.1371/journal.pone.0250821 (PMC8081210; doi:10.1371/journal.pone.0250821)
Supplement: Attachment — Submitted filename: ResponseToReviewers_final.docx [file pone.0250821.s002.docx]

**Manuscript PONE-D-20-06389**

**Response to Reviewers**

Dear Gabriele Fischer,

Thank you for giving us the opportunity to submit a revised draft of the manuscript “Health determinants among refugees in Austria and Germany: a propensity-matched comparative study for Syrian, Afghan, and Iraqi refugees” for publication in PLOS ONE.

We are grateful for the valuable comments we received from you and Reviewer#1. We have incorporated most of the comments made by you and the Reviewer. Those changes are highlighted within the manuscript by tracked changes. Below, in blue, we explain how we have incorporated the comments in the revised version of our paper. All page numbers and line numbers refer to the clean copy of the revised manuscript.

**Editor’s and Reviewer’s Comments to the Authors:**

(1) **Editor Comments**

The manuscript covers an important topic, however more details are required towards the item “self-rated health (SRH)”, which is the key variable: At least the differentiation between somatic or psychiatric health is required; significant literature is published on the high prevalence of psychiatric disorders in refugees/asylum seekers originated from the countries you investigate. More precise information on the addressed questions towards “health” needs to be provided in order to be able to interpret the results – eg affective disorder highly influence the self-perception and can bias given results.

**Author response:**

Thank you for pointing this out. We have provided the exact wording and the possible answers for SRH in the two surveys (pg. 7, ll. 147-150), and added information regarding the validity of SRH (pg. 23, ll. 455-456). We also discuss the interpretation of SRH (pg. 23, ll. 456-470). A precise differentiation of the explanatory power of SRH is not possible, as our data suggest that both somatic and psychiatric health are highly correlated with SRH. Previous research tends to suggest that among AS&R, mental health problems have a somewhat stronger impact on SRH than physical health.

Also information needs to be provided how you counted for the different literacy rates eg in Afghanistan in general, and specifically between men/women – how could you assure that all participants in your target group understood the questions; you covered a certain group of AS & R regarding educational background, where you need to comment specifically on the significant difference between Germany and Austria regarding low level education, which includes the item “no answer” with 43% in Germany (table 1).

**Author response:**

We agree with the reviewer’s assessment, and have provided additional information about the data collection of the two surveys to clarify the data quality and the sources of misunderstanding (pg. 21, ll. 403-419). Moreover, we have added a figure to illustrate the educational differences in Germany and Austria by nationality and gender, including a differentiation between “no answer” and “low level of education" (S1 Fig). As described in the original manuscript, we have calculated weights adjusting for education (applying these weights only slightly affected the results). Our models were adjusted for nationality, education, and sex; and we integrated “education” as a matching variable into the propensity score matching. Finally, we discussed potential bias by education and measures to minimize educational bias as applied in the IA-BAMF-SOEP and ReHIS (pg. 22, ll. 427-444).

This could also refer to the issue, how the questions were addressed: how many items in your questionnaire; how did you correct for interrater-reliability, considering the different applied languages; how many persons interviewed, what was the time range in the performed interviews – again psychiatric morbidity influences the response rate & content of understanding. Was the instrument tested for validity?

**Author response:**

Thank you for this suggestion. We have provided additional information about the questionnaires (including online links to the questionnaires, pg. 6-7, ll. 123-145) and discussed the impact of the different ways the data were collected (CATI interviews in Austria and CAPI interviews with interviewers in Germany; more questions and more time spent in the interviews in the IAB-BAMF-SOEP Survey than in the ReHIS; differences in the languages and the translation; and possible bias due to morbidity or literacy) (pg. 21, ll. 403-419; pg. 23, ll. 454-455; pg. 23, ll. 460-470). Both the ReHIS and the IAB-BAMF-SOEP Survey were pretested. While higher rates of non-response among individuals with lower levels of education and poor health, as discussed in the literature, are likely, they cannot be reconstructed by us. Similarly, as the languages used interfere with the country of origin (Syrians and Iraqis were interviewed in Arabic, Afghans mainly if Dari-Farsi), we are not able to disentangle possible differences between languages on the one hand and country of origin on the other. We do not classify interrater-reliability as a problem in our analysis and data because the analyzed information are based on the respondents’ self-disclosure.

The literature referral towards # 33,35 is not helpful for the readership –a detailed information on the questionnaire is required in the methods section;

**Author response:**

As suggested, we have included detailed information on the questionnaires in the methods section (pg. 5, ll. 96-114) and added additional literature (pg. 5, ll.106 and 114).

also the IRB Nr should be added.

**Author response:**

We appreciate this comment, but our study was exempt from IRB review as we only used de-identified data.

Headline & legend to figure 1 is missing.

**Author response:**

Thank you for pointing this out. The headline and the legend for figure 1 have been included (pg. 15, ll. 306-309).

In a re-submission the manuscript requires a re-structure: detailed information from tables must not be replicated in the text; the abstract requires a structured scientific presentation; limitations need to emphasized clearly.

**Author response:**

As suggested, we have dropped detailed information from the tables (especially information on confidence intervals) in the results section (pg. 10-13). We have adopted the presentation of the abstract (pg. 2), and we have emphasized the limitations (pg. 20-23).

(2) **Reviewer#1 Comments**

Reviewer #1: This is an interesting and timely manuscript. It has good implications for health policies in Germany. Please see my feedback below:

**Author response:**

Thank you.

Pg. 3 line 67 what do you mean by the latter? It is not clear. You may say post-resettlement context or in host country or destination country.

**Author response:**

Thank you for pointing this out. We have clarified that we mean “destination country” (pg. 3, l. 62).

Line 68-69 is hard to follow. Please reword the sentence.

**Author response:**

As suggested, we have reformulated this sentence to make it easier to understand (pg. 3, ll. 63-65).

Pg. 3 line 71 spell out GDP

**Author response:**

As suggested, we have spelled it out (pg. 4, ll. 66-67).

Pg. 4 line 78 now just say GDP in this paragraph.

**Author response:**

As suggested, we have used the abbreviation in this paragraph (pg. 4, l. 73).

Pg. 4 line 78 please briefly explain what EU-28 is

**Author response:**

Thank you. We have briefly explained what the EU-28 is (pg. 4, ll. 74-75).

Pg. 4 line 93 spell out SRH and determinants of what? You can say determinants of health among AS&R

**Author response:**

As suggested, we have spelled out SRH at this point in the text (pg. 4, l. 89), and thereafter use the abbreviation SRH. Moreover, we have clarified that “determinants” refer to determinants of health among AS&R (pg. 4, l. 89).

There should be at least a paragraph explaining your theoretical framework, which I believe is the determinants of health.

**Author response:**

We think this is an excellent suggestion. We referred to the WHO’s “Social Determinants of Health” framework, and have explained this framework briefly (p. 7, ll.152-157).

Pg.4 line 95 do you mean differences in health outcomes?

**Author response:**

Thank you for pointing this out. Yes, we mean differences in health outcomes, and have inserted this term into the manuscript (pg. 4, l. 89).

Pg. 5 line 99 can you tell a little bit more about IAB-BAMF-SOEP-Refugee Survey and ReHIS ? What kind of questions do they include?

**Author response:**

Thank you for this important suggestion. As we mentioned above, we have provided information on the questions included in the two surveys (pg. 6-7, ll. 123-145).

Pg. 5 line 126 please spell out ISCED as well

**Author response:**

Thank you. We have spelled out ISCED when first mentioned (p. 7, l. 163).

Pg. 17 line 348, please explain what period effect means.

**Author response:**

Thank you for pointing this out. We have added a detailed explanation of what “period effect” means (pg. 20-21, ll. 396-402).

Pg. 18 line 359 it is unfortunate that even though you have access to these data sets, your analysis was limited to certain variables for the comparison reason.

**Author response:**

We agree that this is an important suggestion, but the small set of variables was necessary due to the explorative and comparative character of our study. Subsequent studies could employ additional variables.

I think the manuscript would benefit from proofreading, which could greatly improve the flow of the paper.

**Author response:**

Thank you for pointing this out. The manuscript has been proofread.

(3) **Reviewer#2 Comments**

Reviewer #2: No comments from my side as the plosone online submission system is too complicated to interact in a meaningful way and I have no capacity to take time for it. The paper is interesting and I have a few minor comments but cannot submit them here.

**Author response:**

Thank you. We appreciate the effort you took to read our manuscript, and we regret that it was not possible to submit the minor comments.
