# Supplementary material for: Health determinants among refugees in Austria and Germany: A propensity-matched comparative study for Syrian, Afghan, and Iraqi refugees
Source: PLoS One. 2021 Apr 28;16(4):e0250821. doi: 10.1371/journal.pone.0250821 (PMC8081210; doi:10.1371/journal.pone.0250821)
Supplement: Attachment — Submitted filename: ResponseToReviewers_SecondRevision_final.docx [file pone.0250821.s003.docx]

**Manuscript PONE-D-20-06389**

**Response to Reviewers**

Dear Joel Msafiri Francis,

First of all, we appreciate that you agreed to act as Academic Editor as Gabriele Fischer, who was originally assigned to handle our submission, was unavailable for the second round of revisions.

Thank you for giving us the opportunity to further improve our revised draft of the manuscript “Health determinants among refugees in Austria and Germany: a propensity-matched comparative study for Syrian, Afghan, and Iraqi refugees” for publication in PLOS ONE.

We are grateful for the valuable comments we received from you and Reviewer#2. Reviewer#1 had no further comments and recommended to publish the revised daft. We have incorporated most of the comments made by you and Reviewer#2. Those changes are highlighted within the manuscript by tracked changes. Below, in blue, we explain how we have incorporated the comments in the revised version of our paper. All page numbers and line numbers refer to the clean copy of the revised manuscript.

(1) **Reviewer#1 Comments on the revised manuscript**

Reviewer #1: The manuscript has been greatly improved and all my comments have been addressed. I recommend this paper for publication.

**Author response:**

We highly appreciate the recommendation of Reviewer#1 to publish our manuscript.

(2) **Reviewer#2 Comments on the revised manuscript**

Reviewer #2: “105 migration, educational, and employment biographies of AS&R, as well as on their reasons for 106 fleeing, the routes they took, and their personality traits and attitudes [34,35]."
Comment: Personality traits are a scientifically clearly defined issue, to be diagnosed/documented only by experts and/or validated standard questionnaires, else results are at best dubious- discuss…

**Author response:**

Thank you for this objection. In the description of the data, we refer to the information provided by the data holder (reference #35: Brücker/Rother/Schupp, 2018). Presumably, the validity of the results on personality traits might be discussed (particularly when surveying refugees). Since our analyses do not include personality traits, an in-depth discussion would probably go beyond the scope of this paper. Subsequent studies are very welcome to take up and to discuss this dimension. However, to avoid misunderstandings, we have changed “personality traits” to “personality” (l. 109).

“The authors confirm that some access restrictions apply to the data underlying the findings presented here, therefore these data cannot be shared publicly.”
Comment: The authors mention that data details- that are relevant- are published somewhere else, which in my understanding might be against Plosone requirements- For me it is ok, if they are mostly available for checkup, but please summarise at least shortly.

**Author response:**

Thank you for pointing this out. All relevant data are within the manuscript; the references relate to further details about the studies that are not directly related to our analyses, results and discussion. Unfortunately, there are legal restrictions on sharing a (de-identified) dataset. The data cannot be shared publicly, but are available upon request. As requested by PLOS ONE, we have provided the respective DOIs (doi:10.5684/soep.iab-bamf-soep-mig.2016; doi:10.11587/7LX1BD) to the Editors. The anonymous IDs of the respondents selected for the current study are provided upon request.

"For reasons of comparability, the current study is restricted to AS&R who are Syrian, Afghan,
118 or Iraqi nationals aged 18-59 years who immigrated between 2013 and 2016. These three
119 nationalities have made up a large share of the asylum seekers in Europe in recent years,
120 especially in Austria and Germany [1]. Our sample comprises 2,854 respondents in Germany
121 and 374 in Austria." Why the difference ? is this equivalent to the relative differences in the countries subpopulation sizes ? How is the gender balance ? Is it reflecting the one in the population of the refugees or locals ?

**Author response:**

Thanks for that comment. The different case numbers are based on the different sample sizes of the two surveys. We have tried to include information about the maximum number of persons of each survey, resulting in an unbalanced sample with regard to host country. Additionally, the sample is unbalanced with regard to gender (as we have described in line 225 and in Table 1). The gender balance does not reflect the one of refugees or locals. In our analyses, we adjusted for sex to compensate for the imbalance. We have mentioned the problems of imbalance (ll. 435).

And at least a short description of the recruiting strategy and % of those who refused should be given.

**Author response:**

Thank you for pointing this out. We have added a short description of the recruiting strategies (the IAB-BAMF-SOEP-Refugee Survey 2016 included a random sample based on the German Central Register of Foreign Nationals; ReHIS is an interim survey of a panel) and the respective % of refusal (ll. 102, and ll. 111).

"A short cognitive test was also administered."
Comment: Which one ? by experts or (medical) lay persons ? This point must be discussed …

**Author response:**

Thanks for this suggestion. We have added details about the cognitive test (ll. 135). Since our analyses do not include these results, we would like to dispense with a discussion of the implementation and the results of this test.

I do not understand why the else very precise researchers did use an amateur approach to mental health- there are accepted STANDARD scales and questionnaires in that field---the mental health part might be useless though not central to the study…but mental health in refugees and also experiences of violence and torture that are also not included are crucial questions in refugee health, influence perceived health, and have not been covered properly or at all unfortunately--- Also, we do not know, who was actually “certified” (medical findings) sick which might create a different situation then a healthy population or only self "reports" that might equally reflect distress in general..…
I recommend that the authors mention that issues in the limitations, maybe with the excuse that the focus was on perceived health and mainly on the host country health care system in general, the data with the exception of this limitations are interesting and should be published…

**Author response:**

Thank you for pointing this out. We have added a brief explanation of the limitations concerning the measurement of mental health (ll. 483) and emphasized that our focus was on self-rated health (ll. 471).

Finally, there are a lot of data and an excellent handbook of UNHCR (by Kirmayer) on health models, and idioms of distress on the background of culture based illness behavior, that are highly relevant for the groups explored and for the cultural differences in self-reported health, but not considered at all… again this should be at least discussed, as it cannot be fixed anymore at this stage….

**Author response:**

Thanks for this suggestion. Cultural differences in self-rated health are relevant. However, these might be less relevant when comparing two host countries as we have done. We have mentioned this in the discussion (ll. 494) and recommend that subsequent studies include cultural differences.
